# Economically viable electromechanical tensile testing equipment for stretchable sensor assessment

**DOI:** 10.1016/j.ohx.2024.e00546

**Published:** 2024-06-21

**Authors:** Ardi Wiranata, Witnadi Dardjat Premiaji, Widya Kartika, Burhan Febrinawarta, Zebing Mao, Hifni Mukhtar Ariyadi, Nyayu Aisyah, Ryan Anugrah Putra, Kevin G.H. Mangunkusumo, Muhammad Akhsin Muflikhun

**Affiliations:** a Department of Mechanical and Industrial Engineering, Universitas Gadjah Mada, Jalan Grafika No. 2, Yogyakarta 55281, Indonesia,; b Vocational School (D4) of Heavy Equipment Management and Maintenance Engineering, Universitas Gadjah Mada, Gedung TILC, Blimbing Sari, Caturtunggal, Depok Sleman Yogyakarta, 55281, Indonesia; c Faculty of Engineering, Yamaguchi University, Yamaguchi 755-8611, Japan; d Transmission and Distribution Department, PLN Research Institute, Jl. Duren Tiga Raya No.102 Jakarta, 12760, Indonesia

**Keywords:** Conductive material, Electromechanical properties, Soft material, Stretchable sensor, Tensile test

## Abstract

The growing interest in soft robotics increases the demand for stretchable sensors. The high performance of stretchable sensors depends much on the linearity, reliability and hysteresis of the stretchable conductive materials. In the applications of conductive materials such as in dielectric elastomer actuators, a stretchable conductive material should maintain the conductivity while sustaining large and multiple cycles of stretch and release tests. To understand the stretchable electrode quality, researchers should perform an electromechanical test. However, researchers require a high investment cost to use a professional type of electromechanical tensile test. In this research, we proposed an economically viable version of the Do-it-yourself (DIY) electromechanical tensile test (EMTT) to resolve the high investment cost problems. The DIY-EMTT is based on the Arduino-nano module. We integrate the load cell, displacement sensor, motor linear stage and DIY resistance meter. We can use the DIY mechanism to suppress the instrumental cost from thousands to hundreds of dollars. Furthermore, we provide a step-by-step guide to build the DIY-EMTT. We expect our DIY-EMTT to boost stretchable sensor development in soft robotics.

## Graphical abstract

**Figure:**
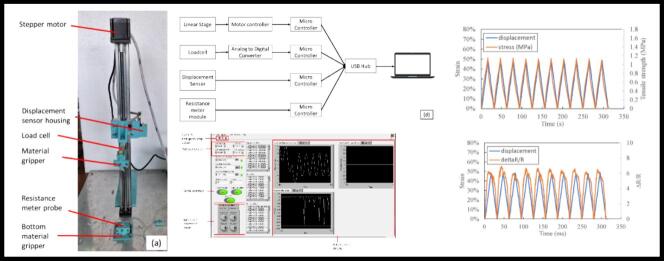

**Specifications table**

**Table:** 

Hardware name	Do-it-yourself (DIY) Electromechanical tensile test equipment
Subject area	Engineering and materials science
Hardware type	Measuring physical properties and in-lab sensors
Closest commercial analog	Universal tensile testing machine integrate with an electrical property measurement device for soft and stretchable conductive material
Open source license	Creative Commons Attribution-ShareAlike 4.0 International License (CC BY-4.0)
Cost of hardware	$173.28
Source file repository	https://doi.org/10.17632/s2mm5btmy5.1

## 1 Hardware in context

Soft robotics is a novel advanced technology in robotics [1]. Recently, soft robotics has gained much attention from researchers in the areas of health care [2], [3], industrial [4], aero space [5], and robotics sectors [1], [5]. Soft robotics consists of two major parts, including sensors and actuators [6], [7]. Some popular soft actuators are pneumatic artificial muscles [8], [9], soft manipulators [10], Dielectric elastomer actuators [11], and soft pumps [12]. The challenge in soft robotics is to produce a high-accuracy movement [5]. In this case, soft actuators combined with high-performance stretchable sensors are a solution for high-precision movement monitoring in soft robotics.

There are types of stretchable sensors [13], including resistance-type sensors [7] and capacitance-type sensors [14]. Each sensor has its characteristics. For example, the resistive sensor has a higher gauge factor, lower fabrication cost, faster fabrication time, and easier measurement method than the capacitance sensor [15]. On the other hand, the strength point of the capacitance sensor includes better linearity, less hysteresis in certain strains, and higher repeatability than the resistive sensors [15]. From the overview, the sensor usage choice lies in the user's decision upon the final application.

Although resistive and capacitive type sensors have their own characteristics, their performance depends much on the quality of the stretchable electrode. High-quality stretchable electrodes should be able to maintain the material's conductivity while sustaining large and multiple cycles of stretch-release [16]. To understand the stretchable electrode quality, novel equipment to measure both mechanical and electrical properties simultaneously is required. In the previous research, Wiranata et al. [6] proposed an electromechanical tensile test for stretchable sensors. This equipment is open source and can be accessed by any researcher worldwide. However, the price of the equipment is relatively high and the hardware's basic operating system has already been predefined by the equipment manufacturer. For example the linear stage motor was from Sigmakoki, the motor controller of the sigmakoki has already had a predefined function to ease the programmer to do the device integration. In this research, we proposed a novel version of the do-it-yourself (DIY) electromechanical tensile test (EMTT). The DIY-EMTT is based on the arduino-nano. We integrate the load cell, displacement sensor, motor linear stage and DIY resistance meter. We provide step by step to build the DIY-EMTT. We expect our DIY-EMTT can boost the development of stretchable sensor in soft robotics.

## 2 Hardware description

**Fig. 1:**
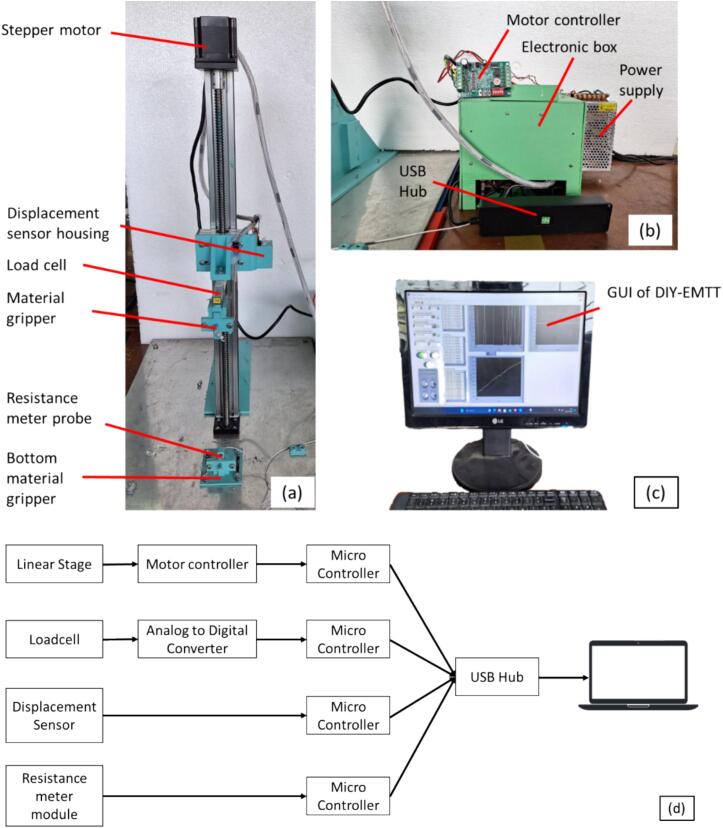
Electromechanical tensile test equipment. a. Linear stage for tensile test equipment, b. controller box and data acquisition, c. GUI for the electromechanical tensile test equipment, d. schematic connection of the electromechanical tensile test equipment.

This section describes the equipment used to build the DIY-EMTT. The equipment is aimed to integrate mechanical tensile testing and electrical property testing. This equipment includes an integrated loadcell module, stepper motor, distance sensor, and resistance meter. All of these modules are integrated using LabVIEW GUI to ease the user. Fig. 1 depicts the overall integration of the electromechanical tensile test equipment. Fig. 1a shows the electromechanical tensile test equipment, Fig. 1b shows the controller and data acquisition box, and Fig. 1c depicts the GUI to control all the equipment. Fig. 1d describes the integration of modules in the electromechanical tensile test equipment. Data from all of the sensors is captured using our GUI. All of the modules are commercially available in the marketplace. We used the module as it is. The material gripper used in this equipment is similar to our previous design [6].

### 2.1 Electrical tester module

**Fig. 2:**
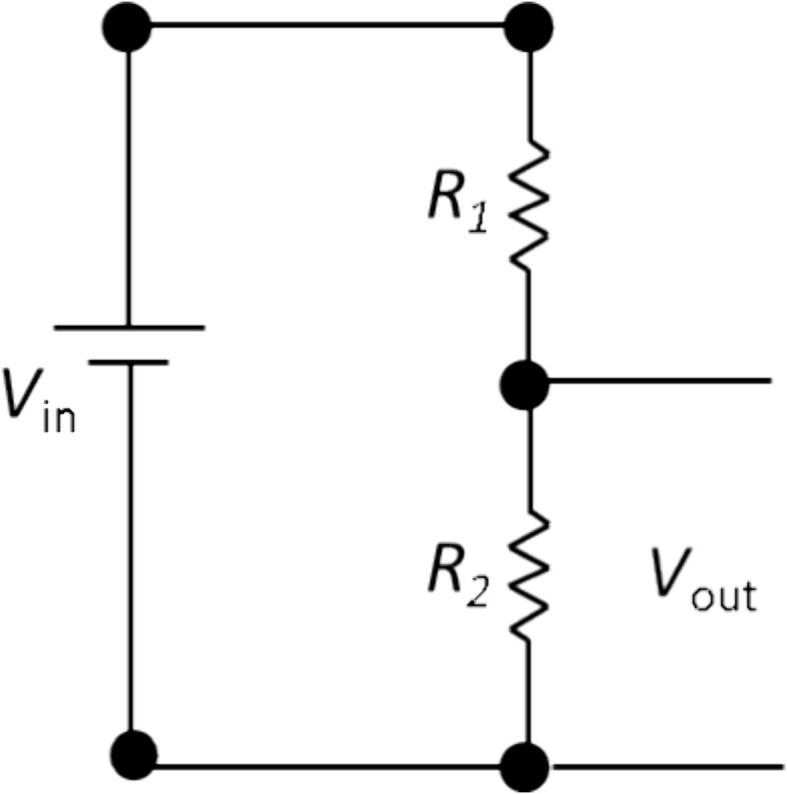
Schematic of simple voltage divider.

In this research, we created a simple DIY resistance meter module. The resistance meter module works by implementing a potential divider mechanism. A potential divider is a circuit that reduces voltage from a sensor to the required level of *V*_out_ [17]. In principle, the equation of the voltage divider is presented in the Eq. (1). The basic schematic of the simple voltage divider is depicted in Fig. 2. Based on Eq. (1), the concept of voltage divider can also be used to predict an unknown resistance.

(1) $$V_{\mathrm{out}}=\frac{R_{2}}{R_{1}+R_{2}}V_{\mathrm{in}}$$

**Fig. 3:**
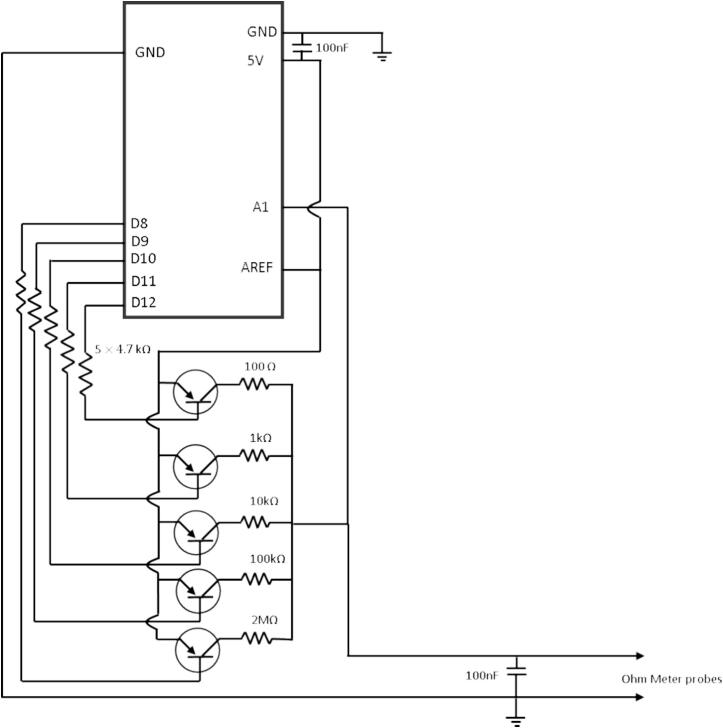
Multiple voltage divider schematic for resistance meter.

We combine the voltage divider with Arduino to calculate the unknown resistance. The challenge is the measurement of higher unknown resistance using this schematic. If the unknown resistance of the circuit is much higher than the known resistance, the resistance measurement is inaccurate. To solve this problem, we create another simple schematic to keep the known resistance value closer to the known resistance. To create this circuit, we required some electronics parts, including a PNP transistor (2SA1015), 100nF ceramic capacitor, 4.7 kΩ resistor, 2 MΩ resistor, 100 kΩ resistor, 10 kΩ resistor, 1 kΩ resistor, 100 Ω resistor, 330 Ω resistor. We also need a microcontroller (Arduino) to control this circuit. The schematic diagram of the system is presented in Fig. 3. In principle, the schematic allows the user to automatically adjust the nearest value of the known resistor to predict the unknown resistor accurately (the Arduino code is also available in the Supplementary material). The accuracy of this voltage divider ranged from 1 % to 2 %. This accuracy depends much on the tolerance of the resistor that is used as the known value resistor. Then, we used a magnetic probe to integrate this resistance meter module to the tensile testing equipment. The magnetic probe was a soft flexible test wires with magnet from aliexpress.com. This magnetic probe test wire was used as it is. Fig. S1 shows the magnetic probe (Fig. S1 available in the Supplementary material uploaded in Mendeley data).

### 2.2 Linear stage module

**Fig. 4:**
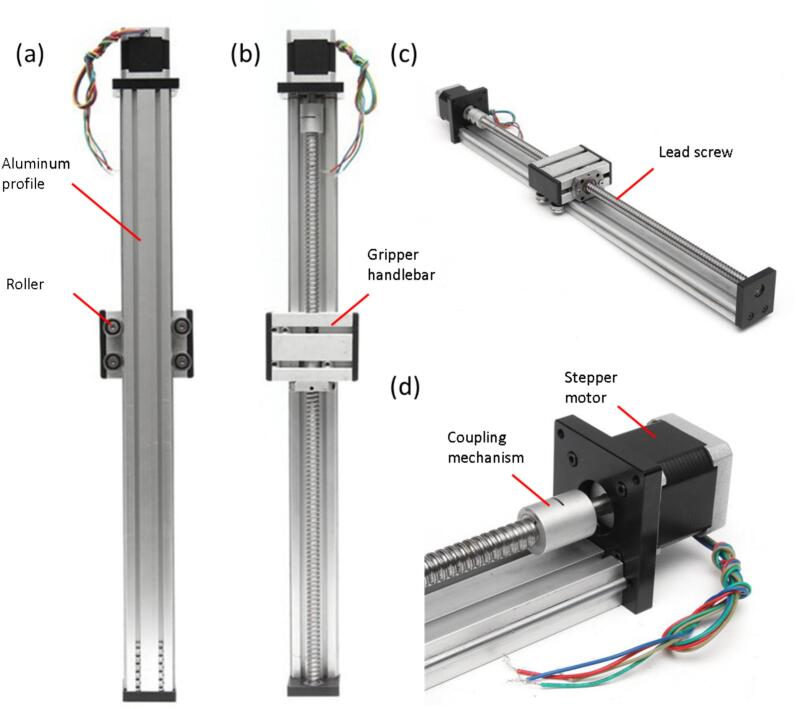
Linear stage module. a. back view of the slider, b. front view of the slider, c. side view of the slider, d. motor coupling mechanism.

The linear stage module used in this research was bought in a pre-assembly state. The linear stage module consisted of an aluminum profile with a length of 500 mm. This aluminum profile was the guiding rail of the slider. The linear movement was motorized by Nema 23. We used a lead screw to convert the rotational movement to the linear movement. To control the motor stepper, we used a TB6560 motor driver. TB 6560 has some features, including the synchronizing step of half step, 1/8 step, and 1/16 step. This feature allows us to create a slow tensile motion according to the ASTM and JIS standards. The figure of the pre-assembled linear stage is presented in Fig. 4. In principle, the linear stage consists of an aluminum profile of 20x40mm as the main rail (Fig. 4b). The stepper motor is coupled with the lead screw using the usual type coupling mechanism (Fig. 4d). We used four steel rollers, as shown in Fig. 4a, to ease the gripper handle bar to move vertically. We mount the material handler to the gripper handlebar (Fig. 4b). The 3D design model is available in the Supplementary information.

### 2.3 Proximity sensor module

We employ a proximity sensor of VL53L0X to monitor the linear stage movement. Basically, we can also measure the movement of the linear stage by calculating from the pulse transmitted to the motor stepper. However, these methods can cause a complicated program. The complicated program can induce motor lagging, leading to the non-smooth movement of the linear stage. In this case, the use of VL53L0X has benefits, including preventing the motor movement lagging and a simpler programming algorithm. VL53L0X is a platform that allows us to measure distance using infrared pulses. The measurement process consists of sending-receiving the reflected infrared and calculating the timing of the sending and receiving signals. According to the datasheet in the marketplace, this sensor can measure distances up to two meters. However, the measurement capability depends significantly on several conditions, such as the surface reflectance, field of view, and ambient temperature. In general, the VL53L0X can measure accurately up to 60 cm in length. Our DIY-Electro mechanical tensile test (DIY-EMTT) has a maximum displacement of 50 cm. We expect the VL53L0X to be the best choice for our applications.

### 2.4 Load cell module

**Fig. 5:**
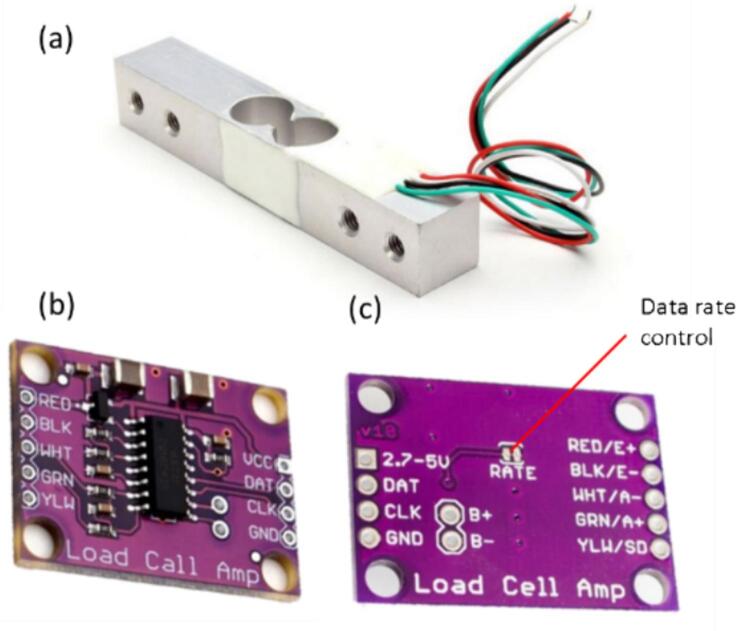
Load cell module. a. loadcell, b. front view of HX711, c. back view of HX711.

We used a low-cost loadcell module to measure the strength of the material in the DIY-EMTT. The load cell module and the HX711 module are presented in Fig. 5. Since we are working with soft materials, we chose a load cell with a load rating of 10 kg. This load cell can be changed according to the researcher's requirements. For the analog-to-digital converter module, we used the HX711. This HX711 has two data transfer modes of 10 Hz and 80 Hz. We can adjust this data transfer mode by connecting or disconnecting small wires at the back of the HX711, as shown in Fig. 5c.

### 2.5 Graphic user interface (GUI) of tensile tester

**Fig. 6:**
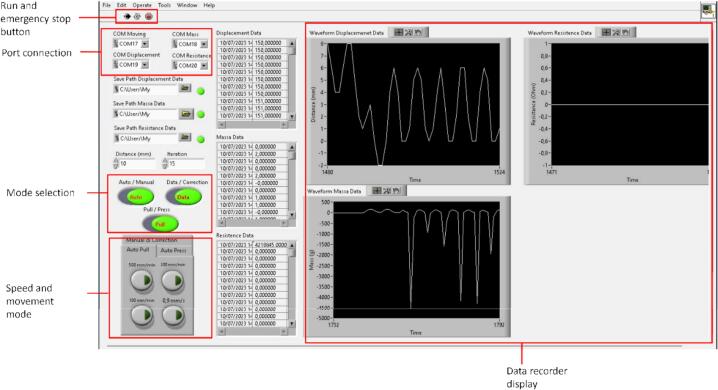
Graphic user interface (GUI) for the DIY-EMTT.

The GUI eases the researchers to operate the DIY EMTT. Researchers can easily define any experimental variable during the stretch and cyclic tensile tests. We create GUI with the Labview platform. Labview platform has two versions: the professional version and the community version. Both of these versions have the same capability. Fig. 6 shows the overall GUI of the DIY-EMTT. The graphic monitoring section in this GUI shows some information, such as displacement, resistance, and load. This GUI measures stress and displacement in grams and millimeters, respectively. Strain and tensile strength can be converted manually from raw data using spreadsheet software.

This system integration aims to simultaneously measure stretchable electrodes' mechanical and electrical properties. We provide a step-by-step process to build a low-cost DIY EMTT. We expect this equipment to be used widely in developing stretchable conductive materials for soft sensors, soft actuators, and soft electric circuits. Our tools are useful for researchers interested in the following research subjects:

• *Soft and stretchable sensors*: High-performance soft and stretchable sensors require completely stretchable electrodes. Ideal stretchable electrodes should be able to preserve their electrical conductivity while sustaining large deformations and durability for millions of cycles. This equipment helps researchers to test the durability of the stretchable sensors and simultaneously test the mechanical hysteresis characteristics of the material.
• *Soft actuators*: High accuracy movement of soft actuators requires low hysteresis materials. In some cases, soft actuators are combined with stretchable sensors to monitor the movement of the actuators. Our equipment can acquire the quality of soft materials in terms of hysteresis at high cycle conditions. Our equipment can also be further modified to test the durability of the embedded sensors in the soft actuators (in terms of delamination possibility, hysteresis possibility of sensors and actuators)
• *Engineering of soft materials*: The DIY-EMTT module enables the measurement of material mechanical and electrical properties simultaneously. This equipment enables researchers of soft materials to test the new soft materials' findings regarding electromechanical properties.
• *Laboratory research facility development:* The DIY-EMTT consists of four simple modules, including a linear stage, displacement sensor, load cell module, and resistance meter. All equipment is commercially available in the marketplace. Any researcher can simply assemble all the equipment. The total cost to build and assemble the DIY EMTT is reasonable compared to other professional packages. Since the equipment is fully DIY, the researcher can modify it easily based on their requirement.

## 3 Design file summary, bill of materials and build instruction

To build the DIY-EMTT, we required parts including a linear stage, motor controller, Arduino nano, load cell module, proximity sensor module, and resistance meter module. For the material gripping part, we used our previous design of the soft material gripper part in Wiranata et al. [6] without any modification. Then, we print the material gripper using Creality Ender 3. The design files and bill of material is presented in Table 1, Table 2

**Table 1:** Design file name and summary.

Design file name	File type	Opensource license	Location of the file
Material Holder (magnetic holder, upper side, and bottom side)	CAD files	CC BY-4.0	https://doi.org/10.17632/s2mm5btmy5.1
GUI software for the DIY EMTT to integrate all equipment	Software	CC BY-4.0	https://doi.org/10.17632/s2mm5btmy5.1
Arduino software for loadcell, Resistance meter, displacement sensor and linear stage	Arduino file	CC BY-4.0	https://doi.org/10.17632/s2mm5btmy5.1

**Table 2:** Material and part list of the DIY EMTT.

Part name	Component	Quantity	Cost per unit in ($)	Total cost in ($)	Source	Material Type
Pre-assembled linear Stage (Fig. 1a)	motorized linear stage	1	95	95	https://best.aliexpress.com/	Metal
Motor stepper controller (Fig. 1a)	TB 6560	1	11.39	11.39	https://www.amazon.com/	Metal
Arduino Nano (Fig. 1b)	Arduino Nano	4	8.99	35.96	https://www.amazon.com/	Electronics
Load cell 10 kg (Fig. 1b)	Load cell	1	5	5	https://www.amazon.com/	Metal and Electronics
Gripper (Fig. 1a)	magnetic holder, material handler upper and bottom side	1	3	3	3D printed part	Polylactit Acid (PLA) 3D printing
Magnetic probe (Fig. 1a)	Test Wires with magnet	1	3	3	https://best.aliexpress.com/	Metal and Plastic
HX711 (Fig. 1b)	loadcell driver	1	10.95	10.95	https://www.amazon.com/	Electronics
USB Hub (Fig. 1b)	USB hub		9.99	0	https://www.amazon.com/	Electronics
VL53L0X (Fig. 1a)	displacement sensor module	1	6.79	6.79	https://www.amazon.com/	Electronics
PNP transistor (2SA1015) (Fig. 1b)	electrical component	5	0.25	1.25	https://www.amazon.com/	Electronics
100 nF ceramic capacitor (Fig. 1b)	electrical component	2	0.14	0.28	https://www.amazon.com/	Electronics
4.7 kOhm resistor (Fig. 1b)	electrical component	5	0.06	0.3	https://www.amazon.com/	Electronics
2 MOhm resistor (Fig. 1b)	electrical component	1	0.06	0.06	https://www.amazon.com/	Electronics
100 kOhm resistor (Fig. 1b)	electrical component	1	0.06	0.06	https://www.amazon.com/	Electronics
10 kOhm resistor (Fig. 1b)	electrical component	1	0.06	0.06	https://www.amazon.com/	Electronics
1 kOhm resistor (Fig. 1b)	electrical component	1	0.06	0.06	https://www.amazon.com/	Electronics
100 Ohm resistor (Fig. 1b)	electrical component	1	0.06	0.06	https://www.amazon.com/	Electronics
330 Ohm resistor (Fig. 1b)	electrical component	1	0.06	0.06	https://www.amazon.com/	Electronics

The challenge in the assembly process lies in the resistance module since we have to solder all the electronic parts into one PCB. One mistake in the soldering process can lead to the wrong measurement or the equipment may not work properly. Basically, the soldering process of the resistance meter is straightforward (the schematic diagram is presented in Fig. 3). Once the resistance meter is finished, we can continue to assemble all the modules.

Besides the resistance meter module, the DIY EMTT consists of a linear stage, loadcell, and displacement sensor modules. We first assemble the linear stage module. The linear stage is pre-assembled, as shown in Fig. 4. To turn on and control the linear stage, we connect the motor stepper to the motor controller (TB6560). The stepper motor consists of four cables (red, blue, yellow, and green). The connection of the cable to the TB6560, power supply, and the Arduino nano can be seen in the table Table 3.

**Table 3:** Connection between Stepper motor, motor driver, Arduino Nano and power supply.

	**Stepper motor to TB6560**		
	**Stepper motor side**	**TB6560**	
	Blue	B−	
	Yellow	B+	
	Green	A−	
	Red	A+	
	**TB6560 – Arduino Nano**		
	CW+	D2	
	CLK+	D3	
	CW−	Ground	
	CLK−	ground	
	**TB6560 – power supply**		
	VCC	24 V power supply	
	Gnd	Ground	

Next, we connect the loadcel module to HX711 and Arduino nano. The connection of the loadcell module is described in table Table 4. This connection is similar to our previous research [6]. Then, we assemble the displacement sensor with Arduino Nano. We used a VL53L0X proximity sensor as a displacement in DIY EMTT. The connection of the wiring cable is presented in Table 5. The connection between the loadcell and displacement sensor modules is straightforward; we used the module as it is.

**Table 4:** Connection of Load cell module.

**Load cell to HX711**	
**Loadcell side** **(Cable color)**	**HX711** **(Pin name)**
Red	E+
Black	E−
White	A−
Green	A+
**HX711 to Arduino-Nano**	
**HX711** **(Pin name)**	**Arduino-Nano** **(Pin name)**
GND	GND
DT	A1
SCK	A0
VCC	5 V

**Table 5:** Connection of displacement sensor module.

**VL53L0X to Arduino Nano**	
**VL53L0X**	**Arduino Nano**
VCC	5 V
GND	GND
SDA	A4
SCL	A5

**Fig. 7:**
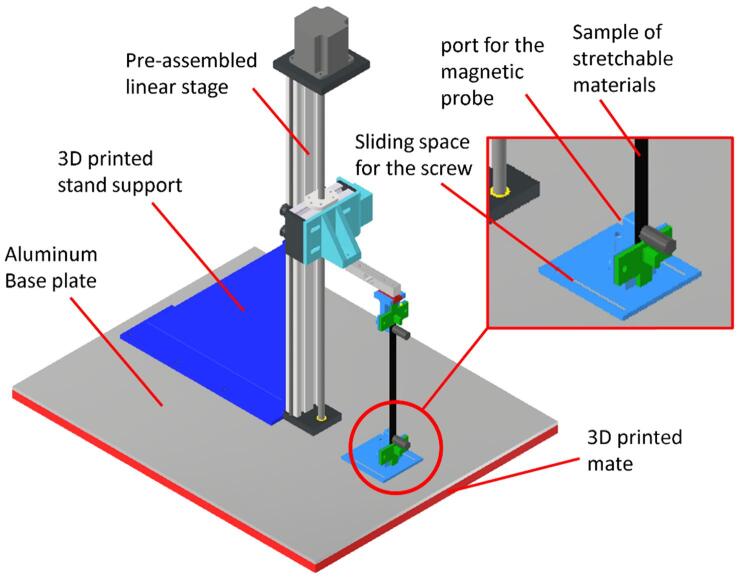
Final assembly of table top DIY EMTT.

After all modules, including the linear stage module, displacement sensor module, loadcell module, and resistance meter module, are assembled. The next process is to integrate the module into DIY EMTT. We can follow the 3D model provided in the supplementary document for the assembly process. The final assembled equipment is presented in Fig. 7. First, we bolt the pre-assembled linear stage to the aluminum base plate. To make the structure rigid, 3D printed stand support was attached to both the pre-assembled linear stage and aluminum base plate (Fig. 7). The loadcell, material gripper, and magnetic probe are assembled as depicted in Fig. 7. The sliding space for screw in Fig. 7 is purposed to adjust the alignment of the bottom gripper and the upper gripper. After the allignment is set, then we secure this sliding space for screw with two bolts.

After assembling all the modules, we put the DIY EMTT on the flat surface (such as table). Then, we connected all the Arduino Nano to the USB hub. This USB hub can merge all the Arduino's USB ports into one single USB connection to the personal computer (PC). When the USB hub is connected to the PC, we can run the DIY EMTT graphic user interface (GUI) (Fig. 6). The operation procedure in detail is presented in the next chapter. In brief, the connection to the PC begins with selecting the port connection. Next, select the place to save the data and hit the run button at the top corner of the GUI. After that, we can operate the DIY EMTT.

## 4 Operations instructions

As mentioned in the previous chapter, the operation begins with selecting the appropriate ports, as shown in Fig. 6. The challenge in this step is remembering all ports suitable to the measuring device. The only way to check the port name is to open the Arduino IDE and then try to read the data one by one. To solve this problem, we can change the name of the port through the NI software. There is one limitation with this method. For example, if we switch the port or randomly plug the USB cable, the port's name may also be switched. This problem also happens in our previous GUI [6]. To manage the limitation, we should not switch the USB port or randomly plug the USB cable. The next step is to select the appropriate folder location to save the data by clicking the save path data tab. Then, we can hit run (in run and emergency button location in Fig. 6). After the run button is pressed, we should ensure that the motor stepper's power supply is turned on. Then, we can select the mode. This software has three different modes: Auto/manual mode, Data/correction mode, and pull/press mode. Auto/manual mode is used to select the operation mode, whether we want to move the linear stepper manual or automate it. The data/correction mode is used to record the data (for data mode) and to tare the sensor measurement (correction mode). Then, pull/press mode is used to choose whether the testing is pulling or pressing, respectively. If we want to tare the sensor reading, we should choose the correction mode and in the tab of manual &correction, we click the correction button. After we tare the sensor reading, we can do a tension test by selecting the mode as follows: 1. put the mode into auto, 2. select the data mode, 3. Activate the pull button, and 4. In the auto pull tab, select the speed that is required. This speed was based on the JIS K 6251 and ASTMD 412. Details about how to operate the equipment in detail is shown in Video S1 (the file is in the repository).

About the speed mode, we have to adjust the stepping mode in the TB6560. Basically, TB6560 has four stepping modes, including 1/1, 1/2, 1/8 and 1/16. These modes allow us to set the speed of the linear stage. For example, if we require a totally slow motion, then 1/16 mode is the best choice. Table 6 presents the TB6560 mode and the speed mode in the GUI. Once we change the mode, we have to adjust the TB6560 manually. By clicking the speed mode in the GUI, the linear stage automatically starts and also records the data. This GUI is customizable to meet the researchers' requirements. For example, a lower speed than 0.8 mm/min is possible by changing the Arduino program and the NI delay of sending the comment to Arduino Nano.

**Table 6:** TB6560 versus GUI speed mode.

TB6560 (step mode)	GUI speed mode	Standard
1/1	500 mm/min	JIS K 6251 & ASTMD 412
1/2	200 mm/min	JIS K 6251
1/8	100 mm/min	JIS K 6251
1/16	0.9 mm/min	N/A

For safety concerns, since the equipment is fully exposed to the environment (Fig. 7) and there is no safety cover to protect the equipment or to isolate the equipment from the user, there is a potential risk of getting caught in the linear stage or scratched by a moving load cell or even hit by fragment of broken material during the tensile testing. To ensure the safety operation of the equipment, users should ensure that the equipment is free from operator’s hands, every users should be in the radius of at least one meters from the equipment and user should use safety glasses during the equipment operation. The use of safety glasses is to protect user from any stray fragment of material during material failure in the tensile testing. By doing this precaution, we expect the safety operation of this equipment can be achieved.

## 5 Validation and characterization

This section shows the application of the electromechanical tensile test equipment to test a stretchable conductive material. The setup of the equipment and the material in the DIY EMTT is depicted in Fig. 7. We tested our equipment for hundreds of cycles to test a stretchable conductive material. Our equipment data rate is stable at 7 data/sec. Our data rate depends greatly on communication between the GUI and the Arduino Nano. In the communication process, the GUI software sends a certain comment to the Arduino, and then the Arduino replies to the comment of the GUI with the data situation. This communication method may take some time. Compared to our previous equipment, this transfer rate is slower. In our previous equipment, the transfer rate was stable at a measuring interval of 100 ms (around 10 data per second). This is possibly due to the communication mechanism between two different platforms. We continuously debug our system to overcome the data rate bottleneck. In our case, the rate of 7 data/sec is enough to measure the mechanical and electrical properties since the tensile speed ranges from 500 – 100 mm/minute. We also did the validation for the electrical tester module and loadcell module using Sanwa LCR 700 and ACIS AD300H respectively. The result of the validation is shown in Fig. S2.

**Fig. 8:**
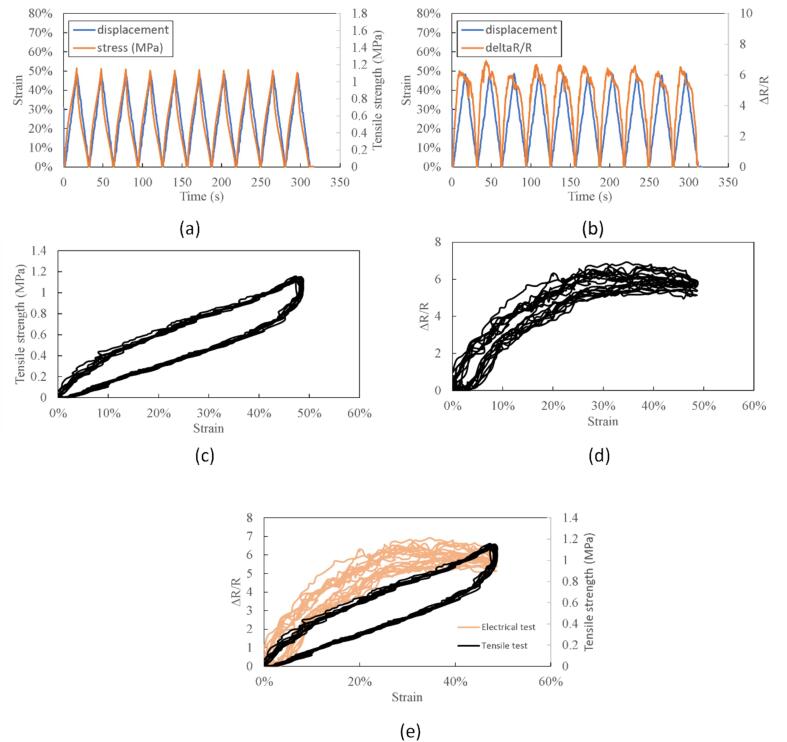
Electromechanical characteristics of conductive materials. a. synchronize data between strain and tensile strength data, b. synchronize data between strain and electrical properties data, c. mechanical characteristics of materials (tensile strength – strain curve), d. electrical properties of the conductive materials (ΔR/R – strain curve), e. comparison of mechanical properties and electrical properties of the conductive materials.

Fig. 8 shows the data of stretchable conductive material taken using our DIY EMTT. We show the first 10 cycles of the material testing result with a speed of 200 mm/min. Fig. 8a and b show the synchronized data results of the tensile and electrical tests. From these figures, we conclude that the data is synchronized well. Next, we further processed the data into a stress–strain curve (Fig. 8c) to show the mechanical characteristics of the soft material during the tensile testing. We also show the electrical characteristics of the conductive soft material in Fig. 8d. As we can see from the Fig. 8d, the gauge factor changed during the stretching process. The material that we used in this experiment was a silicone rubber sheet. We bought the silicone rubber sheet from the marketplace and used it as it is. Then, for the conductive part, we used a conductive grease that is usually used to lubricate the drum in the copy machine. The change in the gauge factor in this conductive material is possibly due to the quality of the conductive materials [6], [7]. Since the data was recorded simultaneously, we can further present the data as shown in Fig. 8e. By presenting these data, the researcher can study the stretchable conductive materials' quality. In the case of our research (Fig. 8e), there is a possible correlation between the mechanical characteristics of the materials and the electrical characteristics material since both results show a hysteresis. This kind of data may differ for different types of materials. By using our DIY EMTT, we expect researchers to easily study the quality of the stretchable conductive materials. The DIY EMTT is totally simpler and cost-effective compared to other devices, and this can ease the researcher to replicate the system.

## 6 Conclusion

This paper describes step-by-step instructions on how to build an electromechanical tensile test equipment (DIY EMTT). We successfully reduced the equipment cost from several thousand dollars to several hundred dollars by using the DIY methods. In principle, the DIY EMTT integrates displacement, resistance tester, load cell, and linear stage modules to simultaneously measure the electromechanical properties of stretchable conductive material. The DIY EMTT is currently designed for soft conductive material with a maximum tensile load of 10 kg. The limit of this equipment lies in the loadcell module limit and the motor stepper module. We can advance the DIY EMTT to measure higher stress values by changing the motor stepper and the loadcell module. There are also shortcomings in this equipment, such as the data rate, which is stable at 7 data/sec. We further debug the system to maximize its data rate. Furthermore, there is also potential vibration from the equipment that can induce unwanted noise in the output data. To overcome this potential problem, we can add an anti-vibration mat under the equipment to reduce the vibration. This anti-vibration mat cost around $83 (including the shipping price) or 47 % of current equipment total price. We expect our DIY equipment to contribute to the development of stretchable sensors and electronics. The low-cost system and easy GUI interface should enable any newcomer researchers in soft robotics to advance the system based on their requirements.

## 7 Ethics statements

We confirmed that our work does not involve any animal or human experiments.

## CRediT authorship contribution statement

**Ardi Wiranata:** Writing – review & editing, Writing – original draft, Conceptualization. **Witnadi Dardjat Premiaji:** Software, Data curation. **Widya Kartika:** Visualization, Investigation. **Burhan Febrinawarta:** Visualization, Investigation. **Zebing Mao:** Validation, Data curation. **Hifni Mukhtar Ariyadi:** Validation. **Nyayu Aisyah:** Validation, Data curation. **Ryan Anugrah Putra:** Investigation, Formal analysis, Data curation. **Kevin G.H. Mangunkusumo:** Investigation, Formal analysis, Data curation. **Muhammad Akhsin Muflikhun:** Writing – review & editing.

## Declaration of competing interest

The authors declare that they have no known competing financial interests or personal relationships that could have appeared to influence the work reported in this paper.

## Appendix A: Supplementary material (Design Files)

All of the supplementary files, including design file and software that mentioned in the manuscript has been uploaded in Mendeley data as a shareable link below (this link also has been mentioned in the main Text): https://doi.org/10.17632/s2mm5btmy5.1.
